# Beyond Morphology: Quantitative MR Relaxometry in Pulmonary Lesion Classification

**DOI:** 10.3390/cancers17203370

**Published:** 2025-10-18

**Authors:** Markus Graf, Alexander W. Marka, Andreas Wachter, Tristan Lemke, Nicolas Lenhart, Teresa Schredl, Jonathan Stelter, Kilian Weiss, Marcus Makowski, Dimitrios C. Karampinos, Daniela Pfeiffer, Gregor S. Zimmermann, Seyer Safi, Hans Hoffmann, Keno Bressem, Lisa Adams, Sebastian Ziegelmayer

**Affiliations:** 1 Department of Diagnostic and Interventional Radiology, School of Medicine, University Hospital Rechts der Isar, Technical University of Munich, 81675 Munich, Germany; alexander.marka@tum.de (A.W.M.); andreas.wachter@tum.de (A.W.); tristan.lemke@tum.de (T.L.); nicolas.lenhart@tum.de (N.L.); teresa.schredl@web.de (T.S.); jonathan.stelter@tum.de (J.S.); marcus.makowski@tum.de (M.M.); dimitrios.karampinos@tum.de (D.C.K.); daniela.pfeiffer@tum.de (D.P.); keno.bressem@tum.de (K.B.); lisa.adams@tum.de (L.A.); s.ziegelmayer@tum.de (S.Z.); 2 Philips GmbH, 22335 Hamburg, Germany; kilian.weiss@philips.com; 3 Laboratory of Magnetic Resonance Imaging Systems and Methods, Ecole Polytechnique Fédérale de Lausanne (EPFL), 1015 Lausanne, Switzerland; 4 Department of Internal Medicine I, School of Medicine, University Hospital Rechts der Isar, Technical University of Munich, 81675 Munich, Germany; gregor.zimmermann@tum.de; 5 Division of Thoracic Surgery, School of Medicine, University Hospital Rechts der Isar, Technical University of Munich, 81675 Munich, Germany; seyer.safi@tum.de (S.S.); hans.hoffmann@tum.de (H.H.)

**Keywords:** lung lesions, T1 mapping, T2 mapping, MR relaxometry, pulmonary nodule classification

## Abstract

**Simple Summary:**

Lung nodules are common and often difficult to classify. Many patients undergo repeated computed tomography (CT), positron emission tomography (PET), or biopsies, all of which have limitations and involve radiation or invasiveness. We investigated whether magnetic resonance relaxometry, which involves taking quantitative measurements of tissue relaxation times (T1 and T2), could help distinguish between benign and malignant lesions. In this prospective study of 64 patients, benign lesions and cancers exhibited distinct relaxation patterns. A classification approach using only T1 and T2 values accurately separated benign and malignant lesions; however, it could not reliably distinguish detailed cancer subtypes. This radiation-free, noninvasive technique may support diagnostic confidence and follow-up decisions.

**Abstract:**

**Background/Objectives**: Lung nodules present a common diagnostic challenge, particularly when benign and malignant lesions exhibit similar imaging characteristics. Standard evaluation relies on computed tomography (CT), positron emission tomography (PET), or biopsy, all of which have limitations. Quantitative magnetic resonance (MR) relaxometry using native longitudinal relaxation time (T1) and transverse relaxation time (T2) mapping offers a radiation-free alternative reflecting tissue-specific differences. **Methods**: This prospective, single-center study included 64 patients with 76 histologically or radiologically confirmed lung lesions (25 primary lung cancers, 28 metastases, 9 granulomas, and 14 pneumonic infiltrates). The patients underwent T1 and T2 mapping at 3T. Two independent readers quantified the mean values for each lesion. The pre-specified primary endpoints were (1) benign versus malignant and (2) primary lung cancer versus pulmonary metastases. **Results**: Significant differences in T1 and T2 values were observed across lesion types. Benign lesions exhibited high T2 values (mean 213.6 ms) and low T1 values (mean 836.6 ms), whereas malignant tumors exhibited lower T2 values (~77–78 ms) and higher T1 values (~1460–1504 ms, *p* < 0.001). Binary classification yielded 95.7% accuracy (sensitivity 93.8% for malignant, specificity 100% for benign) in an internal 70/30 hold-out validation (no external dataset), with consistent performance confirmed by patient-level and nested cross-validation (balanced accuracy ≈ 0.92–0.94). However, malignant subtypes could not be reliably distinguished (*p* > 0.05), and multiclass accuracy was 60.9%. **Conclusions**: Quantitative MR relaxometry allows accurate, radiation-free differentiation of benign and malignant lung lesions and may help reduce unnecessary invasive procedures.

## 1. Introduction

Lung cancer remains the leading cause of cancer-related mortality worldwide, with approximately 1.8 million deaths annually [1]. Despite advances in therapeutic strategies, early detection and accurate characterization of lung lesions continue to be crucial for improving patient outcomes [2]. While low-dose computed tomography (LDCT) has been established as the primary modality for lung cancer screening—reducing lung cancer mortality by 20–24% among high-risk populations [2,3]—the challenge of distinguishing benign from malignant nodules persists, particularly given the high prevalence of indeterminate findings in clinical practice.

Pulmonary nodules are often detected incidentally or during screening, with reported prevalence rates as high as 51% in certain populations undergoing LDCT [4]. Accurate risk stratification and nodule characterization are therefore essential to avoid unnecessary interventions while ensuring timely diagnosis of malignant lesions. Risk prediction models and clinical factors (e.g., size, spiculation, upper-lobe location, age, smoking) allow for estimating malignancy probability [5], and professional guidelines provide management algorithms for incidental nodules [6,7]. However, these conventional parameters are often insufficient to reliably discriminate benign from malignant nodules, particularly for subsolid and small lesions with indolent growth patterns [8].

Magnetic resonance imaging (MRI), long considered suboptimal for lung imaging due to low proton density and susceptibility artifacts, has undergone significant technical evolution in recent years [9]. The advent of ultrashort echo time (UTE) sequences, respiratory gating, and improved coil design now allows high-resolution imaging of the lung parenchyma [10,11].

In healthy lung parenchyma, longitudinal relaxation time (T1) values typically range from 1000–1200 ms, while transverse relaxation time (T2) times are much shorter due to low proton density and rapid dephasing, averaging ~40 ms as measured at 1.5T in previous studies [12,13]. Inflammatory lesions, such as pneumonic infiltrates, exhibit significantly prolonged T2 relaxation times, reflecting increased water content and edema [14]. Malignant lesions, including primary lung cancer and metastases, typically have intermediate T1 values around 1400–1500 ms, often lower than inflammatory or benign lesions [15].

These findings support the concept that magnetic resonance (MR) relaxometry holds promise for the noninvasive classification of pulmonary lesions. The present study investigates whether quantitative T1 and T2 mapping can discriminate between benign and malignant pulmonary lesions and serve as reliable imaging biomarkers to complement existing diagnostic strategies.

## 2. Materials and Methods

### 2.1. Patient Collective and Image Acquisition

This study was designed as a single-center prospective cohort study. Data collection, processing and analysis were approved by the institutional review board (protocol number 692/21S), and informed consent was obtained.

Adult patients (≥18 years) with at least one indeterminate lung lesion on chest CT, such as a solitary pulmonary nodule or inflammatory consolidation requiring further diagnostic clarification, were eligible for inclusion. High-resolution thin-slice chest CT (≤1 mm slice thickness) was required for morphologic reference and lesion localization. In addition, the interval between CT and MRI could not exceed 21 days to ensure temporal consistency of lesion characteristics. Only lesions with predominantly solid components were included; the proportion of solid tissue was assessed on the reference CT (≤1 mm slice thickness) using lung kernel and multiplanar reconstructions. Predominantly solid was determined on thin-slice CT (≤1 mm, lung-window MPR). In line with guideline frameworks (ACR Lung-RADS and the Fleischner Society), management and risk are primarily based on the absolute size of the solid component (e.g., ≈6 mm threshold for part-solid nodules) rather than a percentage of solid tissue. Our ≥50%-solid inclusion threshold was therefore a technical criterion to secure relaxometry SNR and minimize partial-volume effects, not a guideline requirement.

Exclusion criteria included contraindications to MRI (e.g., non-MRI compatible pacemakers or implants), severe claustrophobia, or inability to comply with breath-hold instructions. Previously treated lung lesions (e.g., post-radiation or resected lesions) were not routinely included to avoid confounding tissue changes. MRI datasets with significant motion artifacts affecting image quality were excluded from the final analysis (*n* = 5).

### 2.2. Data Acquisition

All MRI scans were performed on a 3T MRI system (Ingenia Elition X, Philips Healthcare, Best, the Netherlands) using a combination of a 16-channel phased-array anterior coil and an integrated 12-channel phased-array posterior coil. Key sequence parameters (TR/TE, FOV, resolution, SENSE, SPAIR, scan times) are detailed in Appendix A.1. T1 mapping used a MOLLI bSSFP readout (scan time ≈ 15 s) [16], and T2 mapping used a Gradient and Spin Echo (GraSE) readout (echo spacing 11 ms; scan time ≈ 13 s) [17]. A 3D GRE reference with ‘SmartSpeed’ provided isotropic coverage for slice planning [18].

### 2.3. Image Analysis

**Figure 1:**
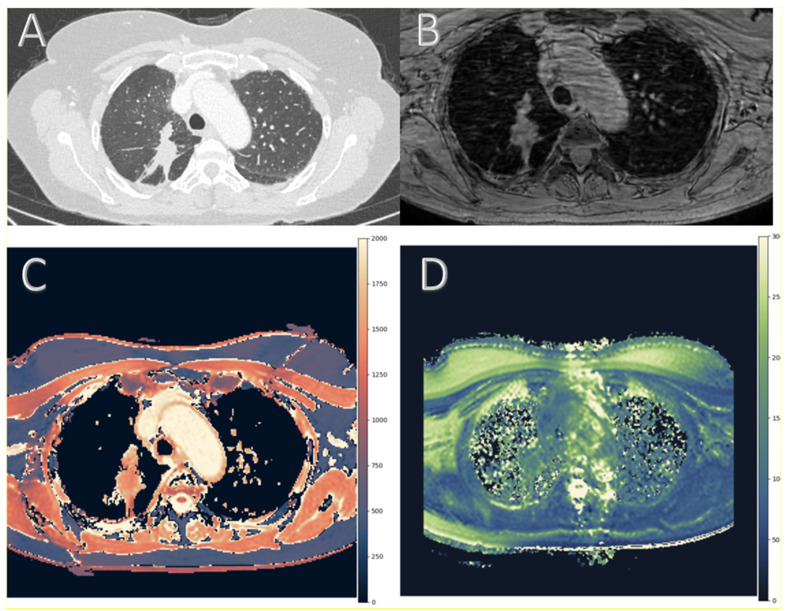
Multimodal imaging of a histologically confirmed primary lung cancer in the right upper lobe. Axial thin-slice chest computed tomography (CT) used for anatomical reference and lesion localization (**A**). Respiratory-gated, AI-accelerated 3D gradient echo image (**B**). T1 relaxation time map and (**C**) T2 relaxation time map (both in ms) (**D**). Panels C and D are color-coded according to International Society for Magnetic Resonance in Medicine (ISMRM) guidelines for magnetic resonance (MR) relaxometry maps, using perceptually optimized diverging color scales [19].

For image analysis, two radiologists with 7 and 9 years of experience in thoracic imaging independently placed regions of interest (ROIs) within each target lesion on both T1 and T2 maps. ROI placement was performed manually using a circular or elliptical shape adapted to the morphology of the lesion. To ensure reproducibility while accounting for lesion size and heterogeneity, each lesion was sampled with up to four ROIs positioned within the most solid and homogeneous areas, preferably on the slice showing the greatest axial extent of the lesion. This cap was pre-specified to balance spatial heterogeneity in sampling (core versus periphery) and reading time. ROI sizes typically ranged from 5 to 20 mm^2^, with adjustments made according to lesion size. For smaller lesions, the ROI was minimized to fit completely within the lesion boundaries to avoid partial volume effects. For larger lesions, multiple ROIs were placed to better represent spatial heterogeneity. ROIs were carefully drawn to exclude necrotic areas, cavitation, bronchi, calcifications, or adjacent vessels and lung parenchyma. Figure 1 illustrates the imaging workflow and generation of relaxometry maps by showing the multimodal imaging of a histologically confirmed primary lung cancer.

Image interpretation was performed under standardized reading conditions on PACS workstations with high-resolution monitors. Readers were allowed to adjust window levels and zoom to optimize lesion assessment. Readers were blinded to histopathology and final diagnosis. CT was used solely for lesion localization, not for classification.

All target lesions were first identified on the available computed tomography scans, which served as a reference for lesion location. Using these CT-defined locations as a guide, each corresponding lesion was then localized on the MRI. Lesions were first searched for on the baseline gradient-recalled echo (GRE) sequences to determine their location within the lung. If a lesion was clearly delineated on these anatomic MRI sequences, its location was then correlated with the corresponding quantitative T1 and T2 relaxation maps for measurement. Only lesions that could be clearly identified on the MRI (in agreement with the CT findings) were included for quantitative analysis and measurement.

Due to excellent inter-reader agreement, the mean of both readers was used for further analysis.

### 2.4. Statistical Analysis

All analyses were performed in Python 3.11 (Python Software Foundation, Wilmington, DE, USA) using SciPy, statsmodels (both NumFOCUS, Austin, TX, USA), and scikit-learn (Inria Foundation, Paris, France). Data preprocessing and visualization were performed with pandas (NumFOCUS, Austin, TX, USA) and Matplotlib (NumFOCUS, Austin, TX, USA). Nonparametric tests, ICC, and machine-learning models are detailed below. Data preprocessing and visualization were performed using the pandas and matplotlib libraries, respectively. All machine learning models, including logistic regression and random forest classification, were implemented with “scikit-learn”.

Primary endpoints were (i) benign vs. malignant and (ii) primary lung cancer vs. metastases. Secondary, exploratory endpoints were (iii) NSCLC vs. SCLC and (iv) a four-class classification (granuloma, pneumonic infiltrate, metastasis, primary lung cancer).

Normality of continuous variables (T1 and T2 relaxation times) was assessed using the Shapiro–Wilk test. As the assumption of normality was not met, nonparametric methods were used for group comparisons. The Kruskal–Wallis test was used to analyze differences in T1 and T2 relaxation times among the four lesion types (granuloma, pneumonic infiltrate, metastasis, and primary lung cancer). When significant group effects were found, post hoc pairwise comparisons were performed using the Mann–Whitney U test. Given the exploratory nature of the study and the small subgroup sizes, no multiplicity adjustment was applied in the main analysis. Exact *p*-values are reported and should be interpreted as hypothesis-generating. In a sensitivity analysis (Appendix A.2), we report additional Bonferroni-adjusted *p*-values. Key findings (notably, the comparison of pneumonic infiltrates and malignant groups for T1/T2) remained significant after adjustment. A *p*-value < 0.05 was considered statistically significant for all tests.

Inter-reader reliability was assessed by calculating the intraclass correlation coefficient (ICC) using the “statsmodels” library, applying a two-way random effects model to assess the absolute agreement between the two readers.

To assess the discriminative potential of T1 and T2 relaxation times in specific diagnostic scenarios, binary logistic regression models were fitted to compare: (i) non-small cell lung cancer (NSCLC) vs. small cell lung cancer (SCLC), and (ii) primary lung cancer vs. pulmonary metastases. In each model, T1 and T2 values were used as independent variables. Model fit was assessed using likelihood ratio tests and pseudo-R^2^ values.

In addition, machine learning models were implemented to classify lesions based on T1 and T2 relaxation times. A binary random forest classifier was trained to distinguish between benign (granulomas and pneumonic infiltrates) and malignant (primary lung cancer and metastases) lesions.

Since several patients contributed more than one lesion (76 lesions from 64 patients), all cross-validation was performed at the patient level using GroupKFold (fivefold) to prevent data leakage. Additionally, a nested cross-validation scheme (outer fivefold and inner twofold) was implemented to optimize the hyperparameters of the random forest classifier. Hyperparameter tuning was performed using a small, predefined grid: number of trees = 100–200, maximum depth = unrestricted or 3, minimum leaf size = 1–2, features per split = 1–2, and class weighting = none or balanced. Balanced-accuracy scoring was used.

For the independent 70/30 hold-out split, 95% bootstrap confidence intervals (5000 resamples) were computed for accuracy, sensitivity, specificity, and balanced accuracy. Calibration quality was evaluated using the Brier score and calibration curves.

A *p*-value of less than 0.05 was considered statistically significant for all statistical tests.

## 3. Results

### 3.1. Study Population

The study population included 64 patients (76 pulmonary lesions), 30 males and 34 females, with a mean age of 64.2 years (SD ± 15.6). The distribution at the patient level was as follows: primary lung cancer (22 patients, 25 lesions); metastases (28 patients, 28 lesions); pneumonic infiltrates (8 patients, 14 lesions); and granulomas (6 patients, 9 lesions). The median interval between CT and MRI scans was 9.0 days (Interquartile Range (IQR): 6.25—17.0).

Regarding lesion types, 28 cases were classified as metastasis, 25 as primary lung cancer, 14 as pneumonic infiltrate, and 9 as granuloma. All metastases and primary lung cancers were confirmed histopathologically. The diagnosis of granulomas was established based on morphologically distinct features, such as calcifications, and through CT follow-up examinations. Of the 25 primary lung cancers, 17 were diagnosed with NSCLC, and 8 patients were diagnosed with SCLC. The histopathological tumor category (pT) of the 25 lung cancer cases was distributed as follows: pT1 (*n* = 2), pT2 (*n* = 2), pT3 (*n* = 18), and pT4 (*n* = 2).

Among the 28 pulmonary metastases, the most common primary tumors were rectal carcinoma (*n* = 4), sarcoma (*n* = 4), prostate cancer (*n* = 3), SCLC with pulmonary metastases (*n* = 3), pancreatic cancer (*n* = 2), non-small cell lung cancer NSCLC with pulmonary metastases (*n* = 2), and uterine cancer (*n* = 2). Additional metastases were from renal cell carcinoma (*n* = 1), jejunal carcinoma (*n* = 1), granulosa cell tumor (*n* = 1), choriocarcinoma (*n* = 1), breast carcinoma (*n* = 1), gastric carcinoma (*n* = 1), melanoma (*n* = 1) and cecal carcinoma (*n* = 1).

Inter-reader agreement was quantified using a two-way random-effects, absolute-agreement ICC. The ICC was calculated using per-lesion, per-reader measurements. For each lesion, each reader’s value was the mean of their ROIs. The two resulting per-lesion values (Reader 1 mean and Reader 2 mean) were then used to estimate inter-reader agreement. Due to the excellent agreement (T1 ICC = 0.98; T2 ICC = 0.97), the average of both readers was used for all subsequent analyses. The mean T1 and T2 relaxation times for each lesion type are summarized in Table 1. Granulomas showed the lowest T2 values, while pneumonic infiltrates showed significantly increased T2 relaxation times. Primary lung cancers and metastases showed comparable mean T1 and T2 values. The mean T1 and T2 relaxation values per lesion entity are shown in Table 2.

### 3.2. Comparison of Relaxation Times Between Different Lesion Types

The Kruskal–Wallis test for T1 relaxation times demonstrated a statistically significant difference between lesion types (H = 13.31, *p* = 0.004). Additionally, the Kruskal–Wallis test for T2 relaxation times exhibited a highly statistically significant difference between lesion types (H = 48.79, *p* < 0.001).

Post hoc analysis was performed with Mann–Whitney-U Test to compare T1 and T2 relaxation times between lesion types, including pneumonic infiltrates, metastases, primary lung cancer, and granulomas. Pairwise comparisons were performed without Bonferroni correction.

For T1 relaxation times, significant differences were observed between pneumonic infiltrates and metastases (U = 68.0, *p* < 0.001) and between pneumonic infiltrates and lung cancer (U = 58.0, *p* < 0.001). However, there was no significant difference between pneumonic infiltrates and granulomas (U = 46.0, *p* = 0.30). Additionally, there were no significant differences between metastases and lung cancer (U = 361.5, *p* = 0.84), metastases and granulomas (U = 146.0, *p* = 0.49), or lung cancer and granulomas (U = 132.0, *p* = 0.46).

For T2 relaxation times, significant differences were found in most comparisons. Pneumonic infiltrates differed significantly from metastases (U = 392.0, *p* < 0.001), primary lung cancer (U = 350.0, *p* < 0.001) and granulomas (U = 126.0, *p* < 0.001). Metastases were also significantly different from granulomas (U = 251.0, *p* < 0.001), and primary lung cancers were significantly different from granulomas (U = 224.0, *p* < 0.001). In contrast, no significant difference was found between metastases and primary lung cancer (U = 368.0, *p* = 0.76).

**Figure 2:**
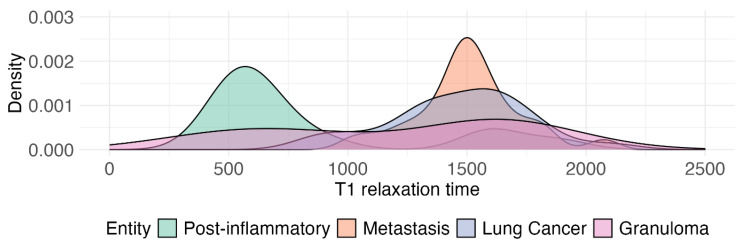
The Kernel Density Estimation (KDE) plot is a graphical representation of the distribution of longitudinal relaxation times (T1) for different lesion entities. The curves represent various lesion types, including metastases, primary lung cancer, pneumonic infiltrates, and granulomas. The *x*-axis of the plot indicates the T1 relaxation time in milliseconds, while the *y*-axis indicates frequency.

**Figure 3:**
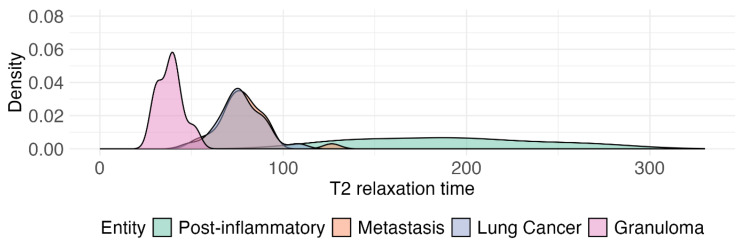
Kernel Density Estimation (KDE) plot visualizes the distribution of transverse relaxation time (T2) relaxation times for the different lesion entities. The *x*-axis of the plot indicates the T2 relaxation time in milliseconds, while the *y*-axis indicates frequency.

**Figure A1:**
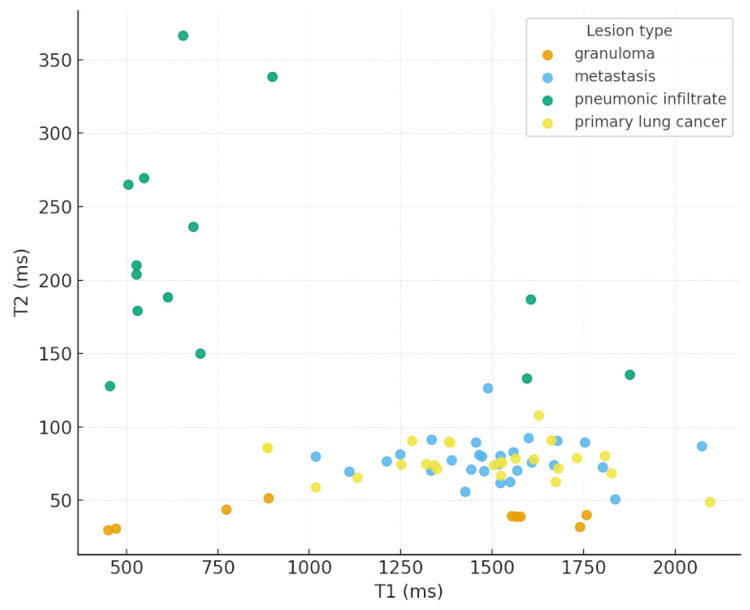
T1 vs. T2 scatter plot of all pulmonary lesions (n = 76).

These results indicate that T1 relaxation times effectively discriminate pneumonic infiltrates from both metastases and lung cancer, while no distinction was evident between granulomas and other lesion types. T2 relaxation times, however, showed significant differences in nearly all pairwise comparisons, underscoring their potential for differentiating between pneumonic infiltrates, metastases, primary lung cancer and granulomas. T2 prolongation reflects increased free-water content and edema in inflammatory lung parenchyma, which explains the marked separation of pneumonic infiltrates from malignant and granulomatous lesions. Kernel Density Estimation (KDE) Plots of the different distributions of T1 and T2 relaxation times for the different lesion types are shown in Figure 2 and Figure 3. A two-dimensional scatter plot showing the relationship between T1 and T2 relaxation times for all lesions is presented in Figure A1.

### 3.3. Primary Lung Cancer vs. Metastasis

A logistic regression analysis was conducted to evaluate whether T1 and T2 relaxation times can differentiate between primary lung cancer and metastatic lesions. The model did not reveal any significant associations. T1 (*p* = 0.53) and T2 relaxation times (*p* = 0.68) both failed to show statistical significance, indicating that variations in relaxation times do not reliably predict the presence of primary lung cancer versus metastases. The overall model fit was poor, with a pseudo R^2^ of 0.007, suggesting that the model explains less than 1% of the variance in the data. The likelihood ratio test yielded a *p*-value of 0.77, further underscoring the model’s inability to make a statistically significant distinction between primary lung cancer and metastases.

### 3.4. Binary Classification: Benign vs. Malignant Lesions

A Random Forest classification model was developed to differentiate between benign and malignant lung lesions using T1 and T2 relaxation times. The dataset included cases classified as granulomas and pneumonic infiltrates, representing benign lesions, and primary lung cancer and metastases, representing malignant lesions. Lesions were grouped into benign and malignant categories, with granulomas and pneumonic infiltrates labeled as benign, while primary lung cancer and metastases were classified as malignant. A binary outcome variable was created, assigning a value of 1 to malignant cases and 0 to benign cases. The final training set included 37 malignant cases and 16 benign cases. The test set consisted of 16 malignant and 7 benign cases. A Random Forest classifier with 100 estimators and a random state of 42 was trained to distinguish between benign and malignant lesions. The data was randomly split into training and testing subsets, with 70% allocated for training and 30% for testing, resulting in 53 cases for training and 23 cases for testing.

To enhance robustness while preventing patient-level leakage, patient-level fivefold cross-validation (GroupKFold) was applied. This approach reduced the likelihood of overfitting and provided a comprehensive evaluation of its predictive power. The Random Forest model demonstrated strong predictive performance in distinguishing between benign and malignant lung lesions. The model’s performance was assessed by measuring its accuracy, precision, recall, and F1-score.

On the test set (*n* = 23), the model achieved an overall accuracy of 95.7%. Sensitivity was 93.8% for malignant lesions, and specificity was 100% for benign lesions (F1-scores: malignant = 96.8%; benign = 93.3%). The feature importance analysis indicated that T2 relaxation times contributed 58.02% to the classification process, while T1 relaxation times accounted for 41.98%.

In the patient-level, fivefold cross-validation (GroupKFold) study, the mean balanced accuracy for benign–malignant discrimination was 0.92 ± 0.12, with a mean sensitivity of approximately 0.98 and a mean specificity of approximately 0.85.

Nested cross-validation (outer fivefold, inner twofold) confirmed stable performance with balanced accuracy of approximately 0.94 ± 0.07 and overall accuracy of approximately 0.95 ± 0.06.

On the independent 70/30 hold-out split, the accuracy was 95.7% (95% bootstrap confidence interval [CI] ≈ 0.87–1.00), with a Brier score of ≈0.03, indicating good calibration and limited overfitting. The model’s robustness was further confirmed by applying class weighting (“balanced”) to account for the mild class imbalance (53 malignant versus 23 benign cases). This yielded comparable performance metrics (accuracy 95.7%, sensitivity 93.8%, specificity 100%) and confirmed stability against class imbalance.

Exploratory endpoints (NSCLC vs. SCLC; four-class classification) confirmed that malignant subtyping was not reliable with relaxometry alone (all *p* > 0.05; multiclass accuracy ≈ 61%); see Appendix A.3 for details.

## 4. Discussion

Our study highlights the strong diagnostic potential of quantitative MR relaxometry in differentiating pulmonary lesions. In particular, the combination of T1 and T2 relaxation times analyzed using a random forest classifier enabled highly accurate discrimination between benign and malignant lesions. With an overall accuracy of 95.7%, a sensitivity of 93.8% for malignant lesions, and a specificity of 100% for benign lesions, our results demonstrate that relaxometry-derived biomarkers represent a promising non-invasive alternative for the classification of lung nodules. These results suggest that quantitative MR relaxometry could play a significant role in improving diagnostic confidence and reducing unnecessary invasive procedures in clinical practice.

Beyond accuracy metrics, these findings may have important implications for clinical decision making, particularly in the context of lung nodule management. Differentiating malignant lesions from benign findings such as granulomatous or (post-)inflammatory changes presents a common diagnostic challenge in routine thoracic imaging. The integration of quantitative MR relaxometry into the evaluation of pulmonary lesions may provide a promising adjunct to thoracic imaging by combining diagnostic information with a radiation-free assessment. Although the internal performance was high, the results were obtained through patient-level and nested cross-validation, which provides robust internal validation. Nevertheless, external, multicenter validation is essential before clinical application. Our results, along with recent literature, underscore the clinical utility of T1 and T2 relaxation times in differentiating benign from malignant lesions [20,21]. Another key advantage of MRI is the absence of ionizing radiation, which is particularly important in patients undergoing serial follow-up or long-term screening. Recent meta-analyses report that lung MRI can achieve a pooled sensitivity of 87.7% for nodules ≥4 mm and up to 98.5% for nodules ≥8–10 mm while maintaining a low false-positive rate [22]. This capability positions MRI as a robust alternative to CT, particularly in scenarios requiring serial follow-up or long-term screening where cumulative radiation exposure is a significant concern [22,23,24].

A major strength of our approach is the potential complementary diagnostic value of T1 and T2 mapping. T1 relaxometry was particularly effective in differentiating pneumonic infiltrates from malignant lesions (*p* < 0.001 each), while T2 relaxation times showed significant differentiation of all benign from malignant lesion types, underscoring their broad applicability in clinical practice. This dual parameter strategy addresses the limitations of using T1 or T2 alone, as each parameter captures different tissue characteristics–T1 reflects cellular composition, and T2 is sensitive to water content and edema.

However, both our findings and previous literature indicate that T1 and T2 relaxometry alone are not sufficient to reliably subtype malignant lesions, such as distinguishing between NSCLC and SCLC or between primary lung cancer and metastases [25]. This limitation is likely due to overlapping biological features such as cellularity and vascularity between different malignant entities. To overcome this, multiparametric MRI approaches–combining relaxometry with diffusion-weighted imaging (DWI) and dynamic contrast-enhanced (DCE) sequences–have shown promising results in improving both specificity and the ability to characterize tumor biology [26]. From a practical perspective, the full relaxometry workflow was quick. It took approximately 15 s for T1 mapping and 13 s for T2 mapping. ROI placement took about one minute per lesion, and model inference took less than one millisecond per case. This demonstrates the method’s potential for clinical feasibility.

A technical limitation of our study is the use of single-slice T1 and T2 mapping sequences. These 2D acquisitions require precise planning based on anatomical reference scans and may only provide partial lesion coverage, which is particularly problematic for large or morphologically heterogeneous lesions. Furthermore, patients with multifocal disease require multiple separate acquisitions, which increases scan time. Recent advances in 3D volumetric mapping offer promising alternatives by enabling whole-lesion coverage, reducing planning dependency [15]. Additionally, restricting inclusion to lesions with a ≥50% solid component for technical reasons related to relaxometry may bias the cohort toward solid lesions. The generalizability of these results to predominantly subsolid or ground-glass opacity nodules remains to be established in future work.

Although several patients contributed more than one lesion, which could have introduced bias, this was mitigated by performing all analyses using patient-level grouping. Despite robust internal validation using nested cross-validation and bootstrap confidence intervals, the small sample size and single-center design warrant external validation.

In addition to the previously discussed issues of single-slice mapping and the absence of an external test set, our ROI strategy purposefully sampled solid, homogeneous regions to minimize partial-volume effects. However, this approach may underrepresent intralesional heterogeneity. Some granulomas were classified based on imaging and CT follow-up rather than histology, which introduces the potential for misclassification. Additionally, all scans were acquired on a single-vendor 3-T platform without phantom calibration or cross-site harmonization. Breath-hold duration was not standardized, and field-strength generalizability (3-T vs. 1.5-T) was not assessed. We did not benchmark against CT-based risk models (e.g., Brock) or perform cost-effectiveness analyses. Future work will include multicenter, multivendor studies with 3D volumetric mapping, phantom-based harmonization, and head-to-head comparisons with radiologists and CT-based risk models. Nevertheless, MR Relaxometry of pulmonary lesions can be seen as complementary tool to morphological assessment, which could be acquired with CSAI GRE/UTE, to improve early differentiation.

Beyond technical aspects, several study-level limitations warrant emphasis. First, no external test set was used, and the patient cohort was imbalanced; thus, internal metrics may overestimate prospective performance. Second, we did not benchmark the model against radiologist performance or CT-based risk models, so comparative utility remains unknown. Third, costs, availability, and workflow impact were not assessed; lung MR relaxometry is not part of current guideline-driven nodule work-up, and sequence availability/standardization varies between vendors. These factors limit generalizability and require prospective, multicenter validation before clinical translation.

Moreover, technical standardization is a critical challenge for the broader implementation of quantitative MRI. Relaxometry values can vary between scanners, vendors, and sequence protocols, affecting reproducibility and comparability between centers. This is particularly relevant as previous studies have shown that standardization of relaxometry protocols remains a significant challenge [27,28].

## 5. Conclusions

In conclusion, quantitative MR relaxometry with T1/T2 mapping demonstrated encouraging results for discriminating between benign and malignant tumors in this single-center cohort. These results are hypothesis-generating and suggest that relaxometry could complement established imaging techniques for risk stratification. However, whether this approach can reduce the need for invasive procedures or change patient management requires prospective, multicenter validation and head-to-head comparisons with radiologists and CT-based risk models. Real-world implementation will depend on sequence availability, protocol standardization, and workflow and cost considerations.

## Figures and Tables

**Table 1:** Summary of cohort characteristics by lesion type (total of 76 lesions from 64 patients). Patient-level counts per entity: primary lung cancer 22 (25 lesions), metastases 28 (28), pneumonic infiltrates 8 (14), granulomas 6 (9). The interval between computed tomography (CT) and Magnetic resonance imaging (MRI) is given as median days [interquartile range]. Histopathology: Primary lung cancers were histologically classified as non-small cell lung cancer (NSCLC) or small cell lung cancer (SCLC); metastases were further classified by primary tumor origin (with “other” including less common primaries such as renal cell carcinoma, gastric carcinoma, melanoma, etc.). All primary lung cancers and metastases were confirmed histopathologically, while granulomas were diagnosed based on characteristic imaging features and follow-up; IQR = Interquartile Range; SD = Standard Deviation.

Lesion Type (*n* = 76)	Age (Years) Mean ± SD	Sex (M/F)	CT-MRI Interval (Days) Median [IQR]	Histopathology/Origin
Primary lung cancer (*n* = 25)	65 ± 15	7/15	10 [4–17]	NSCLC: 17; SCLC: 8
Metastasis (*n* = 28)	63 ± 14	16/12	10 [7–16]	Rectum: 4, Sarcoma: 4, Prostate: 3, SCLC: 3, Pancreas: 2, NSCLC: 2, Uterus: 2, Others: 8
Granuloma (*n* = 9)	62 ± 15	4/2	9 [7–16]	
Pneumonic infiltrates (*n* = 14)	60 ± 18	3/5	8 [8–9]	

**Table 2:** Longitudinal relaxation time (T1) and transverse relaxation time (T2) by Lesion Type; IQR = Interquartile Range.

Lesion Type (*n*)	Mean T1 (ms)	T1 IQR (ms)	Mean T2 (ms)	T2 IQR (ms)
Primary lung cancer	1463.4	1319.5–1661.5	76.9	71.5–86.0
Metastasis	1504.2	1416.6–1601.8	78.1	70.5–84.0
Granuloma	1197.1	773.0–1577.5	38.4	32.0–40.0
Pneumonic infiltrates	836.6	527.4–849.4	213.6	157.2–257.9

## Data Availability

The data presented in this study are available upon reasonable request from the corresponding author. The data are not publicly available due to privacy restrictions.

## Abbreviations

The following abbreviations are used in this manuscript:

CT	Computed tomography
DCE	dynamic contrast-enhanced
DWI	diffusion-weighted imaging
GraSE	Gradient and Spin Echo
GRE	gradient-recalled echo
ICC	intraclass correlation coefficient
IQR	Interquartile Range
ISMRM	International Society for Magnetic Resonance in Medicine
KDE	Kernel Density Estimation
LDCT	low-dose computed tomography
MOLLI	modified Look-Locker inversion recovery
MR	Magnetic resonance
MRI	Magnetic resonance imaging
NSCLC	non-small cell lung cancer
PACS	Picture Archiving and Communication System
PET	Positron emission tomography
pT	pathological tumor category
ROI	regions of interest
SCLC	small cell lung cancer
SD	Standard Deviation
SENSE	sensitivity encoding
SPAIR	Spectral Presaturation with Inversion Recovery
T1	Longitudinal relaxation time
T2	Transverse relaxation time
TE	Echo time
TR	Repetition time
UTE	ultrashort echo time

## Appendix A

### Appendix A.1. Sequence Parameters

Scanner & coils. All MRI scans were performed on a 3 T system (Ingenia Elition X, Philips Healthcare, Best, The Netherlands) using a 16-channel anterior and 12-channel posterior phased-array coil setup.

T1 mapping (MOLLI, bSSFP readout). TR/TE = 2.4/1.1 ms; flip angle 20°; FOV 300 × 300 mm^2^; in-plane resolution acquired ≈ 2.0 × 2.0 mm^2^, reconstructed ≈ 1.2 × 1.2 mm^2^; slice thickness 5 mm; parallel imaging (SENSE factor 3); nominal scan time ≈ 15 s.

T2 mapping (GraSE). TR 1000 ms; 20 echoes with 11 ms spacing (TEeff ≈ 21 ms); FOV 300 × 345 mm^2^; in-plane resolution acquired ≈ 2.0 × 2.2 mm^2^, reconstructed ≈ 1.0 × 1.0 mm^2^; slice thickness 5 mm; SPAIR fat suppression; parallel imaging (SENSE factor 2.5); nominal scan time ≈ 13 s. The 11 ms echo spacing reflects the vendor-optimized GraSE implementation at 3 T, balancing T2-fit stability, echo-train length, and achievable breath-hold duration in the lung.

Reference scan (3D GRE with AI-accelerated compressed sensing, “SmartSpeed”). TR/TE = 2.7/0.83 ms; flip angle 10°; FOV 320 × 557 × 320 mm^3^; voxel 1 mm isotropic; scan time ≈ 233 s. T1/T2 maps were reconstructed inline and transferred to PACS; mapping slices were planned on the reference scan to ensure lesion coverage, with multiple slices in multifocal disease.

### Appendix A.2. Post Hoc Pairwise Analyses and Multiplicity Adjustment

All pairwise Mann–Whitney U comparisons between the four lesion groups were repeated with Bonferroni correction for multiplicity (m = 6 comparisons; two-sided tests).

T1 relaxation times: Kruskal–Wallis H = 13.31, *p* = 0.004. Significant pairwise differences persisted after Bonferroni adjustment for pneumonic infiltrates vs. primary lung cancer (U = 58.0, pBonf = 0.0039) and pneumonic infiltrates vs. metastasis (U = 68.0, pBonf = 0.0040); all other T1 pairs were non-significant after correction.

T2 relaxation times: Kruskal–Wallis H = 48.79, *p* = 1.45 × 10^−10^. After Bonferroni adjustment, all pairs remained significant except metastasis vs. primary lung cancer (U = 368.0, pBonf = 1.000; pRaw = 0.755). Pneumonic infiltrates vs. primary lung cancer (U = 0.0, pBonf = 0.000002), pneumonic infiltrates vs. metastasis (U = 0.0, pBonf = 0.000001), pneumonic infiltrates vs. granuloma (U = 126.0, pBonf = 0.000493), granuloma vs. primary lung cancer (U = 224.0, pBonf = 0.000088), granuloma vs. metastasis (U = 251.0, pBonf = 0.000063).

### Appendix A.3. Exploratory Analyses

#### Appendix A.3.1. NSCLC vs. SCLC (Binary)

Logistic regression using T1 and T2 relaxation times as predictors (NSCLC n = 17; SCLC n = 8) showed no significant association of either parameter with histologic subtype: T1 *p* = 0.88, T2 *p* = 0.35; likelihood-ratio test *p* = 0.45; pseudo-R^2^ = 0.19. Native relaxometry alone did not enable reliable NSCLC–SCLC discrimination.

#### Appendix A.3.2. Multiclass Classification (Granuloma, Pneumonic Infiltrate, Metastasis, Primary Lung Cancer)

A Random Forest classifier trained on T1/T2 values with a stratified 70/30 split achieved 60.9% test accuracy. Benign classes were classified with perfect precision/recall (F1 = 1.0 for granulomas; F1 = 1.0 for pneumonic infiltrates), whereas performance for malignant classes was lower (metastases: precision 42.9%, recall 37.5%, F1 = 0.40; primary lung cancer: precision 44.4%, recall 50.0%, F1 = 0.47). Cross-validation yielded 60.5% ± 12.7% mean accuracy. Conclusion: T1/T2 relaxometry supports robust benign–malignant separation, but is insufficient for fine-grained malignant subtyping in this cohort.
